# Gymnosperm glandular trichomes: expanded dimensions of the conifer terpenoid defense system

**DOI:** 10.1038/s41598-020-69373-5

**Published:** 2020-07-27

**Authors:** Jose M. Celedon, Justin G. A. Whitehill, Lufiani L. Madilao, Joerg Bohlmann

**Affiliations:** Michael Smith Laboratories, University of British Columbia, Vancouver, BC V6T 1Z4 Canada

**Keywords:** Plant sciences, Ecology

## Abstract

Glandular trichomes (GTs) are defensive structures that produce and accumulate specialized metabolites and protect plants against herbivores, pathogens, and abiotic stress. GTs have been extensively studied in angiosperms for their roles in defense and biosynthesis of high-value metabolites. In contrast, trichomes of gymnosperms have been described in fossilized samples, but have not been studied in living plants. Here, we describe the characterization of GTs on young stems of a hybrid white spruce. Metabolite and histological analysis of spruce GTs support a glandular function with accumulation of a diverse array of mono-, sesqui- and diterpenes including diterpene methylesters. Methylated diterpenes have previously been associated with insect resistance in white spruce. Headspeace analysis of spruce GTs showed a profile of volatiles dominated by monoterpenes and a highly diverse array of sesquiterpenes. Spruce GTs appear early during shoot growth, prior to the development of a lignified bark and prior to accumulation of terpenes in needles. Spruce GTs may provide an early, terpene-based chemical defense system at a developmental stage when young shoots are particularly vulnerable to foliage and shoot feeding insects, and before the resin duct system characteristic of conifers has fully developed.

## Introduction

Gymnosperms and angiosperms diverged approximately 300 million years ago^1^. Conifers (Coniferales) represent a large group of gymnosperms with significant ecological and economic roles in many forest ecosystems and forest-dependent industries. Among others, conifers contribute substantially to global carbon sequestration, soil and water conservation, and forest biodiversity. They provide large volumes of valuable wood, fiber and chemical materials for a variety of traditional forest-products and modern bioproducts industries^2^. Conifers have adapted to a wide range of extreme environments where they have evolved unique chemical and physical defenses to deter the attack of insects and pathogens^3,4^. Terpenoid metabolites are a major component of conifer oleoresin defense systems that are produced and stored in specialized secretory structures such as resin ducts, resin blisters, and resin cells. These terpenoid accumulating structures are located on the inside of various organs and tissues and are abundantly present in the stem xylem, phloem and cortex. As an internal defense system, they require tissue damage by, for example, insect feeding, oviposition or pathogen colonization, to release terpenes as deterrents or toxins. Conifers also emit terpenoids as volatiles from young shoots and needles that can attract beneficial insects^5–8^. The emission of terpenes from conifer needles is thought to occur through stomata^7–10^; however, the mechanism of emission and whether other cell-types may contribute remain largely unknown.

Trichomes are typically multicellular structures that protrude from the above-ground surfaces of plants and can be divided into two main categories: non-glandular (i.e., hairs) and glandular (i.e., secretory) trichomes. The most distinct feature of glandular trichomes (GTs) is their capacity to biosynthesize, store and secrete a large diversity of specialized metabolites including terpenoids, alkaloids, polysaccharides, and polyphenols^11–14^. The compounds produced by GTs function in plant defense and protection and also provide an important source for traditional and modern bioproducts, such as fragrances^15^, food additives^16,17^, or pharmaceuticals^18^. Glandular and non-glandular trichomes have been extensively characterized in a variety of angiosperms, including characterization of their cellular and subcellular structures, transcriptomes, and metabolomes. Despite earlier reports of uncharacterized “pubescent” structures in gymnosperms and bryophytes^19,20^, the description of conifer fossils containing trichome-like structures^21,22^, and the mention of spruce “Drüsenhaare” (i.e. GTs) in some of the classical literature on conifer resins^23^, to our knowledge, there are no reports on a detailed characterization of trichomes in gymnosperms.

Here we describe the characterization of GTs on the surface of young shoots of interior white spruce, which represents a naturally occurring genetic admix of white spruce (*Picea glauca*), Engelmann spruce (*Picea engelmannii*), and Sitka spruce (*Picea sitchensis*). Spruce GTs are formed prior to bud break and mature as the shoot expands. Histological analysis revealed large intracellular storage compartments and lack of an extracellular secretory cavity. They accumulate substantial amounts of mono-, sesqui- and diterpenes, including methylated diterpenes, which have been significantly associated with insect resistance in white spruce (*P*. *glauca*)^24^.

## Results

### Spruce GTs have a single-cell, globular head with large intracellular storage compartments and begin to develop prior to bud break

**Figure 1:**
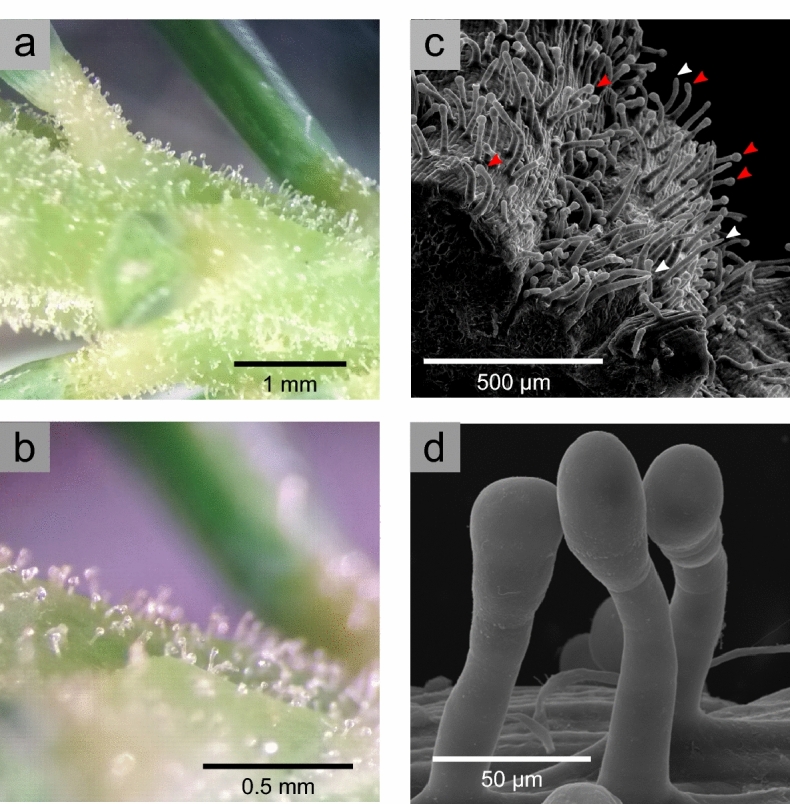
Glandular trichomes (GTs) cover the young, elongating stems of hybrid white spruce (*P. glauca* × *P. engelmannii* × *P sitchensis*, genotype PG29). GTs were imaged with a dissecting microscope **(a, b)**, and by scanning electron microscopy **(c, d)**. GTs cover the entire stem surface, including the pulvini of needles **(a)**. Most trichomes are characterized by a globular head (**c**, red arrows), but some have a narrow terminal cell (**c**, white arrows).

We found trichomes densely covering the surface of the stem axis of young, elongating shoots, including the base (pulvini) of needles, of a hybrid white spruce (genotype PG29) (Fig. 1). This genotype has been used as a reference for spruce genomics^25,26^ . The majority of trichomes showed a globular expansion at their apex suggesting a glandular-type trichome (Fig. 1d). We used scanning (SEM) and transmission (TEM) electron microscopy to investigate these trichomes in more detail. SEM identified two general trichome morphologies, a predominant type with a globular head (Fig. 1c, red arrowheads) and less frequent type without globular head (Fig. 1c, white arrowheads). Trichomes with a globular head represented ~ 90% of trichomes on the surface of fully elongated young shoots. In contrast, on younger and still elongating stems, trichomes without a globular head made up 50% or more of trichomes (Fig. S1). Elongating stems also had small epidermal protuberances which may be the initial phase of trichome formation (Fig. S1). Trichomes without globular heads may represent an intermediate stage of development prior to expansion of globular heads.

**Figure 2:**
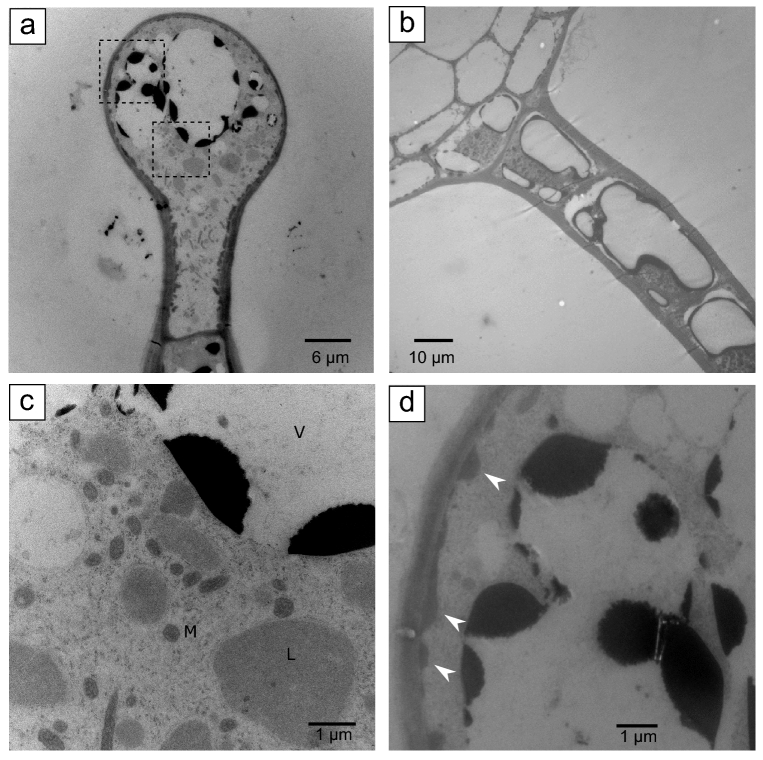
Transmission electron micrographs (TEM) of spruce trichomes. **(a)** TEM imaging of trichome globular heads showed they consist of a single cell with dense cytoplasm and large vacuole-like compartments with electron dense contents stained with osmium. No evidence of an extracellular secretory cavity could be observed. **(b)** TEM images of trichome stalks showed they are composed of multiple cells with dense cytoplasm and vacuole-like compartments with electron dense contents stained with osmium, similar to globular heads. Higher magnification of areas demarked with dashed lines in **(a)** are shown in panels **(c)** and **(d)**. Multiple vesicles with distinct electron density localize next to the plasma membrane all around the globular head (**d**, white arrows). V, vacuole-like; L, leucoplast, and M, mitochondria.

TEM of GT heads showed the head volume filled with dense cytoplasm and several large intra-cellular storage compartments with vacuole-like features and dark electron-dense contents after osmium staining (Fig. 2a). TEM images of trichome stalks revealed a multicellular structure also with cytoplasmic-dense cells, each with compartments having vacuole-like features and dark electron-dense contents after osmium staining, similar to head cells (Fig. 2b). Leucoplasts were found closely associated with the endoplasmic reticulum in head and stalk cells (Fig. 2c). Head cells also had numerous vesicles in close proximity to the plasma membrane at the periphery of the cell. (Fig. 2d, white arrowheads). These vesicles had a distinct electron density, different from the contents of leucoplasts and vacuole-like compartments, and were absent in stalk cells. GTs had an average height of ~ 180 µm (± 27 s.d., n = 5), with stalk cells having a cross-section of ~ 21 µm (± 3.1 s.d., n = 6), and the globular head a cross-section at the widest point of ~ 29 µm (± 2.8 s.d., n = 4).

**Figure 3:**
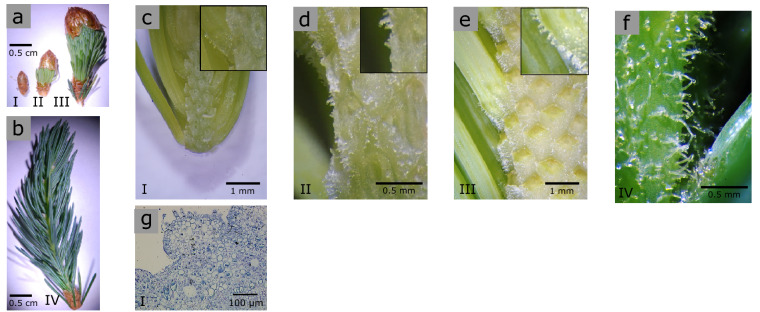
Spruce GTs start developing prior to bud break. Developing shoots were collected at four different developmental stages from the same tree at the same time: swollen bud (**a**, stage I), elongating/flushing stems (**a**, stages II and III) and fully elongated stems (**b**, stage IV) to track the formation and appearance of GT. Trichomes could be observed in all four developmental stages **(c, d, e, f)** including in closed, swollen buds **(c)**. Insets in panels **(c), (d),** and **(e)**, show a closer image of developing GT. Toluidine blue staining of stem sections (500 nm thick) after fixation and embedding in LR white resin, confirmed GT trichomes start forming at the bud stage **(g)**.

To gain a better understanding on the time-course of GT formation, we collected stems at different developmental stages from bud swelling to fully elongated shoots and analyzed them by light microscopy (Fig. 3a,b). We found trichomes at all developmental time-points, including buds that showed small protuberances on the surface of the stem axis before bud break (Fig. 3c,g). At later stages during stem elongation, the developing trichomes became longer and started developing globular heads (Fig. 3d–f).

### Spruce GTs accumulate mono-, sesqui- and di-terpenes metabolites

**Figure 4:**
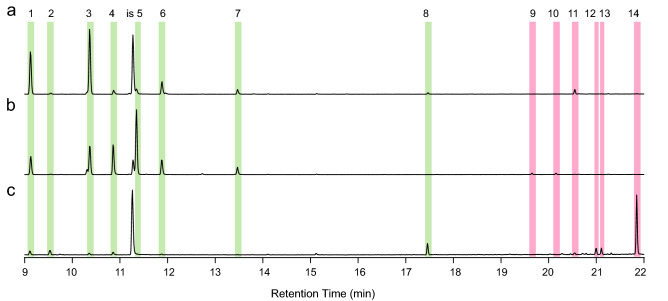
Monoterpenes and sesquiterpenes detected in spruce GT extracts. Shown are total ion chromatograms of metabolite extracts from isolated GT **(a)**, stems after trichomes have been removed **(b)**, and needles **(c)**. The profile of GT was dominated by monoterpenes (green-shaded areas, peaks 1–8) with minor relative amounts of sesquiterpenes (red-shaded areas, peaks 9–14). Stem terpene profiles were also dominated by monoterpenes but with different relative amounts compared to GT. Needle terpene profiles were dominated by the sesquiterpene germacrene-D-4-ol and characterized by minor amounts of monoterpenes. Isobutyl benzene was used as internal standard (is). Mono- and sesquiterpenes identified with authentic standards are peak 1: (-)-α-pinene; peak 3: (-)-β-pinene; peak 4: β-myrcene; peak 7: α-terpinolene; peak 8: bornyl acetate; and peak 14: germacrene-D-4-ol. Peaks that could not be identified are 2, 5, 6, and 9–13. Also see Table S1 for terpene identification.

**Figure 5:**
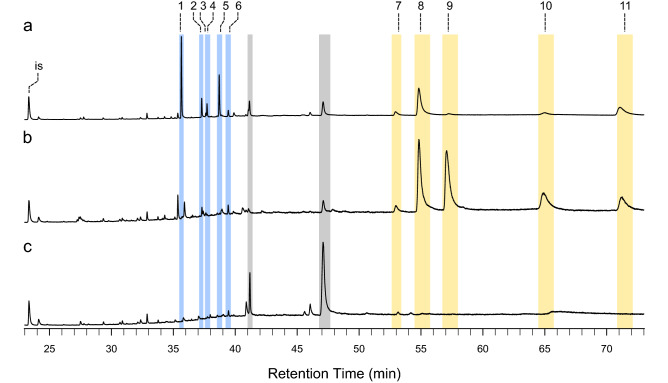
Diterpene resin acids and diterpene methyl esters detected in spruce GT extracts. Shown are total ion chromatograms of metabolite extracts from isolated GT **(a)**, stems after trichomes have been removed **(b)**, and needles **(c)**. GT diterpene profiles contained major peaks of methylated diterpenes (blue shaded areas) including methyl palustrate (peak 1) methyl abietate (peak 4), methyl neoabietate (peak 5). GT also had several peaks of unknown diterpenes with *m/z* 330 (peaks 2, 3, and 6). Stem diterpene profiles were characterized by major peaks of diterpenes resin acids sandaracopimaric acid (peak 7), palustric acid (peak 8), isopimaric acid (peak 9), levopimaric acid (peak 10), and neoabietic acid (peak 11). Needle metabolite extracts only had trace amounts of diterpenes. Eicosene was used as internal standard (is). Methylated diterpenes and diterpene resin acids were identified with authentic standards.

To test if spruce GTs contain terpenoid specialized metabolites, which are prominent in other conifer defensive structures, we isolated trichomes, stems (after trichomes had been removed), and needles and extracted their metabolites with methyl *tert*-butyl ether (MTBE). Gas chromatography-mass spectrometry (GC–MS) of these extracts showed that trichomes contained mono-, sesqui-, and diterpenoids (Figs. 4, 5). The diterpenoids included diterpene methylesters, which are rare in nature. Terpenes present in the trichome extracts included the monoterpenes (−)-α-pinene, (−)-β-pinene, β-myrcene, α-terpinolene, and (−)-bornyl acetate and a sesquiterpene of unknown identity (Fig. 4). Diterpenes in trichomes included methyl-palustrate, methyl-abietate, methyl-neoabietate, sandaracopimaric acid, palustric acid, isopimaric acid, levopimaric acid, and neoabietic acid. Diterpene methylesters occurred naturally in trichomes and were not the result of trimethylsilyl diazomethane derivatization. The GC–MS traces of trichome extracts revealed three peaks of unknown identity with a *m/z* 330 and mass spectra with characteristic fragment ions likely originating from diterpene backbones (Fig. 5 and Fig. S2). Extracts of the young stems without trichomes had a similar terpene profile compared to GTs, but with a lower ratio of methylated to non-methylated diterpenes and lower relative amounts of the unknown *m/z* 330 peaks. Needle terpene profiles at this developmental stage were characterized by low relative amounts of monoterpenes and sesquiterpenes except for germacrene-D-4-ol, which had a higher relative amount in needles and was not detected in trichomes or stems (Fig. 4c). Diterpenes were not detected in needle extracts (Fig. 5c).

**Figure 6:**
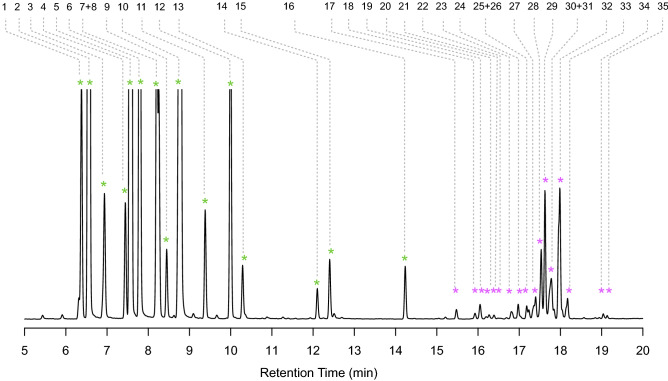
Spruce GTs contain a large diversity of volatile monoterpenes and sesquiterpenes. Isolated GT were collected in a headspace vial and volatiles were sampled with a solid-phase microextraction (SPME) fiber and analyzed by GC–MS using an HP-5 column. GT trichomes volatiles consist mostly of monoterpenes followed by a highly diverse profile of sesquiterpenes. Peaks were identified by a combination of authentic standards, linear retention index calculated with alkane standards, and best library hit using the NIST and Whiley libraries as shown in Table S1.

To investigate profiles of volatile terpenes in spruce GTs in more detail, we performed solid-phase microextraction (SPME) sampling coupled with GC–MS with isolated trichomes. SPME sampling of trichome volatiles revealed a highly diverse profile with more than 35 different mono- and sesquiterpenes (Fig. 6; Table S1). The major peaks corresponding to volatiles detected by SPME GC–MS represented the same monoterpenes observed in the MTBE extracts. More than 16 different monoterpenes were observed in the SPME profiles including camphene, phellandrene, and linalool. SPME GC–MS of sesquiterpenes revealed more than 19 different sesquiterpene compounds.

## Discussion

Conifers are protected by multiple chemical and physical defenses. At maturity, their massive stems are shielded to the outside with a thick bark formed by layers of lignified and suberized cells. On the inside of their stems, the xylem, phloem and bark of mature and young trees contain a reticulate system of resin ducts filled with terpene-rich oleoresin^3,27^. In addition, clusters of stone cells with reinforced cell walls^28^, and layers of polyphenolic cells rich with potentially toxic phenolics^29^ provide the outer stem tissues with protective barriers. While these defensive structures and their chemicals are typical of fully developed stems, they are missing or are only just emerging in young elongating shoots, including the apical shoot. The phenology of a delayed development of these major conifer defenses makes conifers vulnerable to herbivores at the time around bud burst, which coincides with the time when insects pests such as budworms or aphids may be most active^30,31^. The GTs described here in a hybrid white spruce, and their specialized terpene metabolites, may provide a developmentally early protection to young emerging shoots. It appears that GTs had previously been overlooked by us and others in the characterization of conifer defense systems^3,4,29^. Trichomes are mentioned as taxonomic descriptors for a few bryophytes and gymnosperms including GTs in spruce ^19,20,32^, and they have been reported in fossilized samples of ancient gymnosperms^21,22^. Beyond that, we are not aware of a characterization of GTs in conifers, or in any other gymnosperm species. GTs appear to be absent in Sitka spruce (*P. sitchensis*) and are sparsely found in white spruce (*P. glauca*) stems (Fig S3), suggesting that GTs in the hybrid white spruce may originate with the *P. engelmannii* component in the genetic admix. GT may also be present in developing stems of sugar pine (*Pinus lambertiana*), where structures called colleters have been described to secrete their contents on the surface of young stems^33^.

Here we showed that spruce GTs accumulate a complex profile of mono-, sesqui-, and diterpenes, including methylated diterpenes and unknown diterpenes. The GT terpene profile was different from stems and needles and enriched for methylated diterpenes. Diterpene methylesters have been reported in a few conifer species including methyl levopimarate and methyl palustrate in stone pine (*Pinus pinea*), red pine (*Pinus resinosa*) and Scots pine (*P. sylvestris*)^34,35^, and the norditerpene *o*-methylpodocarpic acid in white spruce (*P. glauca*)^24^. Interestingly, higher contents of *o*-methylpodocarpic acid in white spruce bark were associated with resistance to white pine weevil (*Pissodes strobi*)^24^. Diterpene methylesters have not been widely observed in many conifers, which may be due to the common practice of diterpene derivatization with methylating reagents for GC–MS analysis, and they may be more prevalent than currently thought. To our knowledge, diterpenes with *m/z* 330 have not been reported in conifers, and may originate from an additional oxidation of diterpene methylesters (Fig. S2). Such oxidation may be catalyzed by cytochromes P450. P450s of the conifer-specific CYP720B subfamily produce diterpene resin acids^36–38^, while other conifer P450s may insert an additional oxygen in the diterpene backbone.

Conifers typically accumulate terpenes extracellularly in the lumen of resin ducts that are embedded within various tissues throughout the different organs of the tree. To act as a defense, terpenes must be released from resin ducts by tissue damage caused for example by insect feeding or oviposition or as a result of pathogen colonization. In contrast to resin ducts, spruce GTs lack an extracellular secretory space, but appear to accumulate terpenes intracellularly. Two different compartments of the GTs may be involved in terpene accumulation: the large vacuole-like compartments present in GT heads and the numerous small vesicles close to the plasma membrane. Volatile terpenes may be released from intact GTs through the plasma membrane or from herbivore damaged GTs. The vesicles next to the plasma membrane of GT head cells may provide a route for terpene release from intact GTs. Terpenes released from GTs may serve as feeding deterrents or as signals for beneficial insects^6,39^. The spruce GTs increase the surface from which terpenes may be emitted, which in turn may enhance the defensive function of terpene emissions.

Plant trichomes have been extensively characterized in angiosperms^12,14,40^. The present characterization of terpene-producing GTs in spruce provides an opportunity for future comparative studies of GTs in angiosperms and gymnosperms, which diverged ~ 300 million years ago^1^, with potential implications for a better understanding of the evolution of GTs. Formation of glandular-type trichomes in angiosperms involves various different gene regulatory networks^41^, suggesting that GTs evolved more than once and independently in different species. Studies on fossilized conifer tissues from the Late Carboniferous period^21,22^ suggest the possibility that trichomes existed before the radiation event that gave origin to angiosperms. Future transcriptomic studies on spruce GTs may help identify genes responsible for trichome formation in a gymnosperm, and may also lead to the genes responsible for diterpene resin acid methylation.

## Methods

### Plant material

Clonally propagated hybrid white spruce trees (*P. glauca* × *P. engelmannii* × *P. sitchensis*, genotype PG29, 7 years old) were grown outside in 2-gallon pots. Young flushing stems (4–6 cm long) were collected in the spring. Needles were carefully removed from stems by hand and samples were immediately flash frozen for metabolite analysis, or were cut into 2–3 mm long sections with a razor blade and transferred to fixation buffers for SEM or TEM.

### Trichome isolation and metabolite analysis

Trichomes were isolated from frozen stems by gentle brushing with a cooled paintbrush over a mortar containing liquid nitrogen and ground with a pestle^42,43^. To minimize losses of ground trichomes, metabolites were extracted directly in the chilled mortar by adding 2 mL of methyl *tert*-butyl ether (MTBE) with isobutyl benzene (19 µM), and 1-eicosene (12 µM) as internal standards. Upon thawing, the MTBE-trichome mixture was transferred to a GC vial and extracted at 22 °C with shaking for 2 h. The extract was centrifuged at 2,000 × *g* for 2 min to remove insoluble materials, and stored at − 80 °C until GC–MS analysis. Stems (after removing trichomes) and needles were ground in a mortar with liquid nitrogen and extracted with MTBE in a GC vial at 22 °C with shaking for 2 h. Diterpenes were analyzed using an Alltech AT-1000 column (26.6 m × 0.25 mm ID; 0.25 µm film thickness) with Helium as carrier gas, set at 0.6 mL min^−1^ on an Agilent 7,890/7000A GC/MSD triple Quadrupole system equipped with an Agilent 7,683 autosampler. The GC oven temperature program was as follows: 50 °C for 2 min, increase at 5 °C min^−1^ to 230 °C and hold for 20 min. Injection was done in pulsed splitless mode at 250 °C inlet temperature. Mono- and sesquiterpenes were analyzed on an Agilent HP-5 column (5% phenyl methyl siloxane, 30 m × 0.25 mm ID; 0.25 µm film thickness) with Helium as carrier gas, set at 0.8 mL min^-1^ on an Agilent 6890A/5,973 GC/MSD system equipped with an Agilent 7,683 autosampler. The GC oven temperature program was as follows: 40 °C for 4 min, increase at 6 °C min^−1^ to 90 °C, followed by increase at 9 °C min^−1^ to 310 °C and hold for 4 min. Injection was done in pulsed splitless mode at 240 °C inlet temperature. Headspace SPME analysis was performed using a 100 µm PDMS (polydimethylsiloxane) fiber for extraction of volatiles at 40 °C for 10 min and analyzed on an Agilent DB-WAX column (polyethylene glycol, 59.4 m × 0.25 mm ID; 0.25 µm film thickness) with Helium as carrier gas, set at 0.8 mL min^-1^ on an Agilent 6,890/5,973 GC/MSD system equipped with an Agilent PAL RSI 85 autosampler. The GC oven temperature program was as follows: 40 °C for 3 min, increase at 10 °C min^−1^ to 100 °C, followed by increase at 3 °C min^−1^ to 130 °C, followed by increase at 30 °C min^−1^ to 250 °C and hold for 2 min. Injection was done in split mode with a ratio of 3:1 at 250 °C inlet temperature. Headspace SPME analysis was similarly performed in an HP-5 column with Helium as carrier gas set at 0.8 mL min^−1^ on an Agilent 7,890/7000A GC/MSD triple Quadrupole system. The GC oven temperature program was as follows: 50 °C for 2 min, followed by increase at 7 °C min^−1^ to 150 °C, followed by increase at 25 °C min^−1^ to 300 °C and hold for 2 min. Injection was done in split mode with a ratio of 3:1 at 250 °C inlet temperature. Alkane standards were injected using SPME sampling in the DB-WAX and HP-5 columns to calculate linear retention indexes of mono- and sesquiterpenes. Trichomes used for SPME analysis were isolated as described above with the exception that they were not ground. Instead, isolated frozen trichomes were transferred with liquid nitrogen directly from the mortar to a head-space vial placed in dry ice. Upon evaporation of the liquid nitrogen, vials were immediately closed and analyzed by SPME GC–MS. Chiral analysis was performed on an Agilent Cyclodex-B column (30 m × 0.25 mm ID, 0.25 µm film thickness) with Helium gas as carrier set at 0.9 mL min^−1^ on an Agilent 6,890/5,973 GC/MSD system. The GC oven temperature program was as follows: 40 °C for 1 min, increase at 3 °C min^−1^ to 80 °C, followed by increase at 25 °C min^−1^ to 240 °C, and hold for 5 min. Injection was done in split mode with a ratio of 5:1 at 240 °C inlet temperature. Terpene identification was done by best match to linear retention index reported in the literature; comparison with reference mass spectra from databases of the National Institute of Standards and Technology (NIST) MS library searches (Wiley W9N08L) and relevant literature; and comparison with retention time and mass spectra of authentic analytical standards when available.

### Microscopy

Fresh, non-fixed PG29 GT were observed with a WILD M3B dissecting microscope (WILD, Heerbrugg, Switzerland) and imaged with a Nikon D80 digital camera. A series of focus stack images were created manually and processed with Helicon Focus software. Stem samples for electron microscopy were cut into 2 mm long pieces with a sharp razor blade, and fixed in freshly made 0.1 M sodium cacodylate buffer (pH 6.9) containing 1.25% gluteraldehyde and 2% paraformaldehyde. Samples were fixed under vacuum in a low-temperature microwave (PELCO 3,450 Laboratory Microwave, USA) in 4 cycles of 2 min each. Samples were removed from fixation buffer, washed with 0.1 M sodium cacodylate (pH 6.9) under vacuum, and postfixed with 1% w/v OsO_4_ in 0.1 M sodium cacodylate (pH 6.9). Samples were washed in distilled water and dehydrated under vacuum in an ethanol series until reaching 100% ethanol. Samples used for SEM were transferred into fresh 100% ethanol in ceramic watch glasses, and the ethanol was allowed to evaporate in a fume hood. Samples were stuck to non-conductive adhesive sticky tabs mounted on aluminium SEM stubs (Ted Pella, Inc.), coated with 5 nm gold/platinum at 2.5 kV using a Cressington 208C High Resolution Sputter Coater, and examined with a Hitachi S-2600N Variable Pressure SEM device. The stem surface of young shoots was examined for three samples obtained from different trees. Samples for TEM were gently vortexed once in 100% ethanol to detach trichomes from stems. Stem pieces were removed with tweezers and detached-trichomes were infiltrated with Spurr’s resin in 10% increment steps of 1 h each. Once in 100% Spurr’s resin, trichomes were transferred to blocks with fresh resin, centrifuged at 1,000 *g* for 5 min to place them horizontally at the bottom of the block, and polymerized at 60 °C for 16 h. Silver sections 70 nm thick were obtained with glass knives in a Leica UC7 ultramicrotome and mounted in single slot grids. Mounted sections were stained with 2% uranyl acetate for 10 min, and Reynolds lead citrate for 5 min^44^. Digital images were captured with a Hitachi 7600 TEM (Nissei Sangyo America Ltd.) at 80 kV.

## Supplementary information

Supplementary Information.
