# Supplementary material for: Gymnosperm glandular trichomes: expanded dimensions of the conifer terpenoid defense system
Source: Sci Rep. 2020 Jul 27;10:12464. doi: 10.1038/s41598-020-69373-5 (PMC7385631; doi:10.1038/s41598-020-69373-5)
Supplement: Supplementary file 1 — Supplementary Information. [file 41598_2020_69373_MOESM1_ESM.pdf]

Supplementary File

Gymnosperm Glandular Trichomes: Expanded Dimensions of the Conifer Terpenoid Defense System

Jose M Celedon[1], Justin G. A. Whitehill[1], Lufiani L. Madilao[1], and Joerg Bohlmann[1*]

[1]Michael Smith Laboratories, University of British Columbia, Vancouver, BC V6T 1Z4, Canada

*corresponding author: E-mail: bohlmann@msl.ubc.ca

Table S1: Terpenes identified by SPME GC-MS in spruce GT

Peak No	Compound	LRI $_{lit}$ [a]	LRI $_{exp}$ [b]	LRI $_{exp}$ [c]	Identification
1	α-Thujene	924	926	-	1,2
2	(-)-α-Pinene	932	934	991	1,2,3
3	Camphene	946	951	1025	1,2
4	Sabinene	969	974	1081	1,2,3
5	(-)-β-Pinene	974	979	1067	1,2
6	β-Myrcene	988	989	1124	1,2,3
7	α-Phellandrene	1002	1007	1126	1,2,3
8	δ-3-Carene	1008	1010	1111	1,2,3
9	α-Terpinene	1014	1018	1140	1,2,3
10	β-Phellandrene	1025	1032	1069	1,2,3
11	γ-Terpinene	1054	1059	1203	1,2
12	Terpinolene	1086	1087	1239	1,2,3
13	Linalool	1095	1100	1491	1,2
14	Terpinen-4-ol	1174	1184	1553	1,2
15	γ -Terpineol	1199	1198	1643	1,2
16	Bornyl acetate	1284	1287	1529	1,2
17	α-Cubebene	1345	1351	1431	1,2
18	α -Copaene	1374	1374	1466	1,2
19	Unknown	-	1381	-	-
20	β-Cubebene	1387	1388	1560	1,2
21	Unknown	-	1392	-	-
22	Unknown	-	1398	-	-
23	Unknown	-	1429	-	-
24	Aromadendrene	1439	1441	1675	1,2
25	Unknown	-	1457	-	-
26	Unknown	-	1460	-	-
27	Unknown	-	1473	-	-
28	α-Amorphene	1483	1483	1682	1,2
29	Unknown	-	1491	-	-
30	Unknown	-	1502	-	-
31	γ -Cadinene	1513	1511	1710	1,2
32	δ-Cadinene	1522	1526	1705	1,2
33	Unknown	-	1546	-	-
34	Unknown	-	1655	-	-
35	α-Cadinol	1652	1671	1891	1,2

Peak No: peak number according to retention times on an HP-5 column as shown in **Fig 5**.
[a] LRI $_{lit}$: Retention indexes extracted from (Adams et al., 2007).
[b] LRI $_{exp}$: Determined linear retention index against mixture of n-alkanes (C8-C31) on HP-5 column.
[c] LRI $_{exp}$: Determined linear retention index against mixture of n-alkanes (C8-C31) on a DB-wax column.
Identification by: (1) match to linear retention index reported in the literature, (2) comparison with reference mass spectra from databases of the National Institute of Standards and Technology (NIST) MS library searches (Wiley W9N08L) and relevant literature; and (3) comparison with retention time and mass spectra of authentic analytical standards.

Figure S1

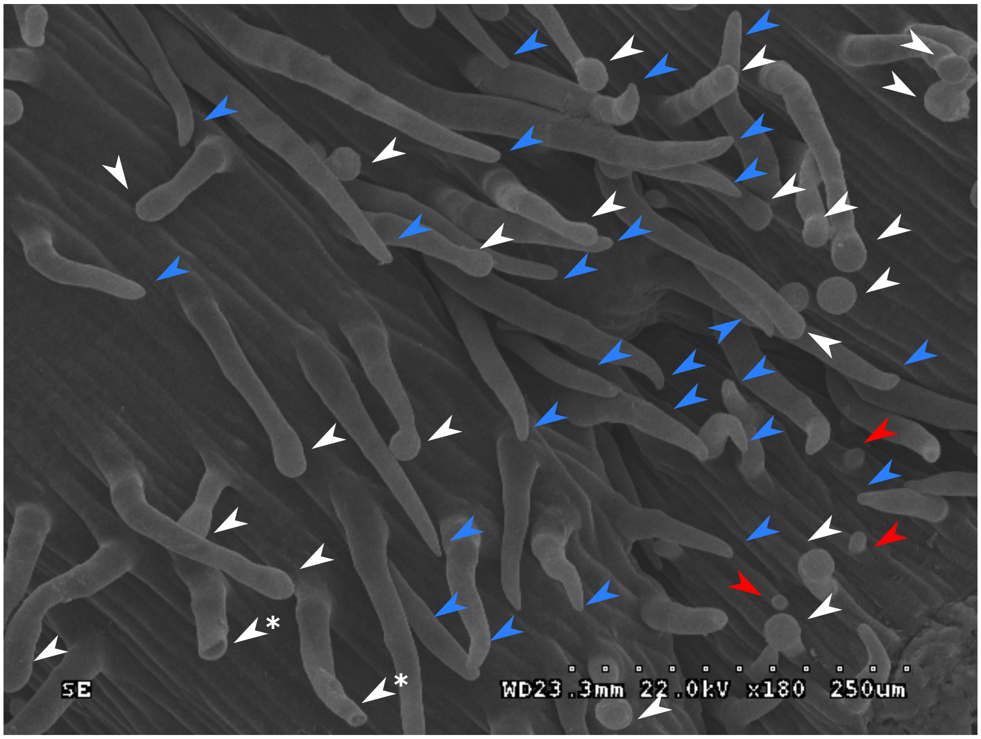

Figure S1: SEM image of glandular and non-glandular type trichomes in PG29 stems. Heads of glandular-type trichomes are indicated by white arrows, non-glandular type trichome tips are indicated by blue arrows and trichomes at early development stages are indicated by red arrows.

Figure S2

a

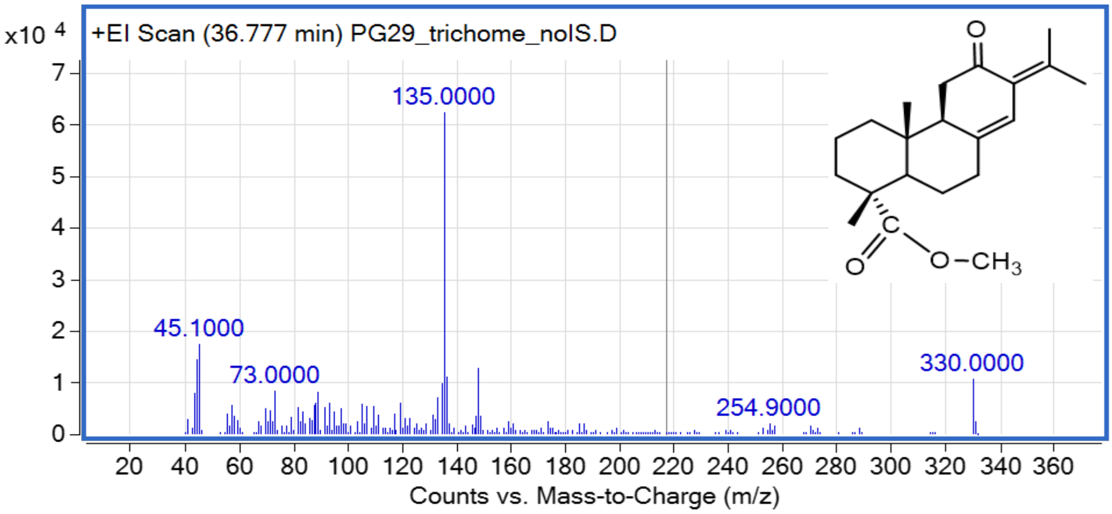

b

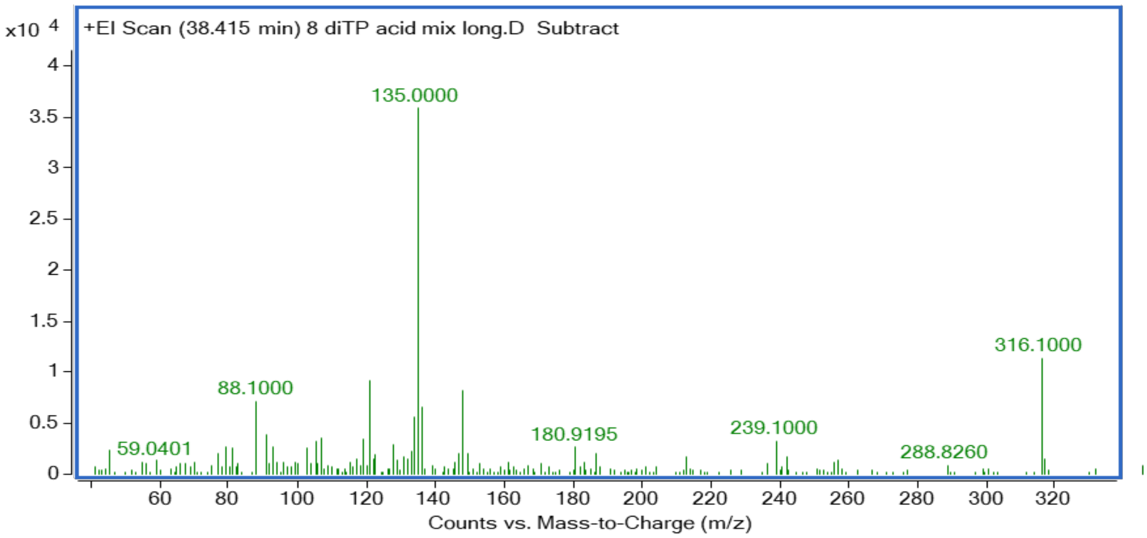

Figure S2. The mass spectra of one unknown compound with m/z 330 has a characteristic ion 135 similar to Neoabietic acid. The mass spectra of one of the unknown compounds with m/z 330 was obtained from underivatized extracts and shown to have a dominant ion of mass 135 (a). The inset in (a) shows a hypothetical diterpene structure with a natural methyl ester group and an additional oxygen which would result in a compound with m/z of 330. For comparison, the mass spectra of derivatized Neoabietic acid is shown in (b).

Figure S3

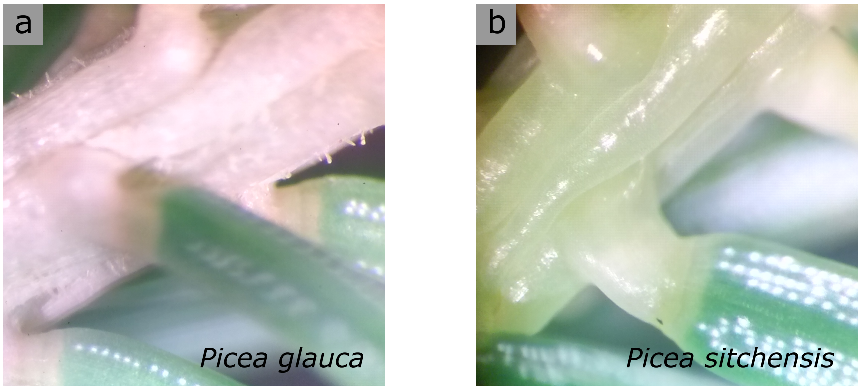

Figure S3. Trichomes are sparsely found in *Picea glauca* stems (a) and completely absent in *Picea sitchensis* stems (b).
